# Machine learning and the nomogram as the accurate tools for predicting postoperative malnutrition risk in esophageal cancer patients

**DOI:** 10.3389/fnut.2025.1606470

**Published:** 2025-06-18

**Authors:** Zhenmeng Lin, Hao He, Mingfang Yan, Xiamei Chen, Hanshen Chen, Jianfang Ke

**Affiliations:** ^1^Department of Thoracic Oncology Surgery, Clinical Oncology School of Fujian Medical University & Fujian Cancer Hospital, Fuzhou, China; ^2^Department of Anesthesiology Surgery, Clinical Oncology School of Fujian Medical University, Fujian Cancer Hospital, Fuzhou, China; ^3^Department of Operation, Clinical Oncology School of Fujian Medical University & Fujian Cancer Hospital, Fuzhou, Fujian, China; ^4^Department of Thoracic Oncology Surgery, First Affiliated Hospital of Fujian Medical University, Fuzhou, China

**Keywords:** esophageal cancer, postoperative malnutrition, machine learning, nomogram, surgery

## Abstract

**Background:**

Postoperative malnutrition is a prevalent complication following esophageal cancer surgery, significantly impairing clinical recovery and long-term prognosis. This study aimed to develop and validate predictive models using machine learning algorithms and a nomogram to estimate the risk of malnutrition at 1 month after esophagectomy.

**Methods:**

A total of 1,693 patients who underwent curative esophageal cancer surgery were analyzed, with 1,251 patients allocated to the development cohort and 442 to the validation cohort. Feature selection was performed via the least absolute shrinkage and selection operator (LASSO) algorithm. Eight machine learning models were constructed and evaluated, alongside a nomogram developed through multivariable logistic regression.

**Results:**

The incidence of postoperative malnutrition was 45.4% (568/1,251) in the development cohort and 50.7% (224/442) in the validation cohort. Among machine learning models, the Random Forest (RF) model demonstrated optimal performance, achieving area under the receiver operating characteristic curve (AUC) values of 0.820 (95% CI: 0.796–0.845) and 0.805 (95% CI: 0.771–0.839) in the development and validation cohorts, respectively. The nomogram incorporated five clinically interpretable predictors: female gender, advanced age, low preoperative body mass index (BMI), neoadjuvant therapy history, and preoperative sarcopenia. It showed comparable discriminative ability, with AUCs of 0.801 (95% CI: 0.775–0.826) and 0.795 (95% CI: 0.764–0.828) in the respective cohorts (*p* > 0.05 vs. RF). Calibration curves revealed strong agreement between predicted and observed outcomes, while decision curve analysis (DCA) confirmed substantial clinical utility across risk thresholds.

**Conclusion:**

Both machine learning and the nomogram provide accurate tools for predicting postoperative malnutrition risk in esophageal cancer patients. While RF showed marginally higher predictive performance, the nomogram offers superior clinical interpretability, making it a practical option for individualized risk stratification.

## 1 Introduction

Esophageal cancer is the seventh most common malignancy globally and the sixth leading cause of cancer deaths (1, 2). Curative resection, which involves tumor removal and lymph node dissection, remains pivotal for improving survival (3). However, these procedures disrupt gastrointestinal anatomy and function, leading to dysphagia, early satiety, and postprandial dumping syndrome, all of which contribute to early postoperative malnutrition (incidence: 44.0–75.7%) (4–8).

Malnutrition in cancer patients is not just a comorbidity but a potentially life-threatening condition. Studies indicate that 10–20% of cancer patients die from malnutrition-related complications rather than the cancer itself (9). After esophagectomy, malnutrition worsens metabolic dysfunction, increases infection risk, prolongs hospital stays, and raises 30-day mortality (8, 10–12). Moreover, malnourished patients have reduced tolerance to adjuvant therapies (e.g., chemotherapy, immunotherapy), further decreasing survival rates and quality of life (13–17).

Current guidelines recommend early enteral nutrition to reduce postoperative malnutrition risk; however, choosing the best delivery method remains clinically challenging (18, 19). Nasojejunal feeding, a minimally invasive method, is usually limited to short-term use (4–6 weeks) due to risks such as tube displacement and aspiration pneumonia (20). In contrast, surgical jejunostomy allows long-term nutritional support but can cause complications like bowel obstruction (21–23). This highlights the need for preoperative malnutrition risk stratification: high-risk patients benefit from prophylactic jejunostomy during esophagectomy, whereas low-risk patients can safely use temporary nasojejunal feeding.

Although predictive models for malnutrition exist in gastric and colorectal cancer cohorts (24–26), no validated tools are available for esophageal cancer’s unique challenges. Moreover, existing studies frequently lack standardized diagnostic criteria. To address this gap, our study is the first to apply the Global Leadership Initiative on Malnutrition (GLIM) criteria—a robust, consensus-based diagnostic system—to assess postoperative malnutrition (27, 28). We aimed to develop and validate accurate predictive models to improve preoperative risk stratification, providing clinicians with interpretable tools for personalized nutritional interventions based on individual risk profiles.

## 2 Methods

### 2.1 Study population and data collection

The development cohort included 1,251 esophageal cancer patients treated at Fujian Cancer Hospital, with data retrospectively analyzed from a prospective database spanning September 2021–January 2025. For external validation, an independent cohort of 442 patients was established from the First Affiliated Hospital of Fujian Medical University, covering March 2022–January 2025. Extracted variables encompassed baseline characteristics, preoperative laboratory values, intraoperative parameters, oncological profiles, and postoperative outcomes.

Inclusion Criteria: patients were eligible for inclusion if they (1) had histologically confirmed esophageal cancer; (2) underwent radical esophagectomy; (3) were aged ≥18 years.

**Figure 1:**
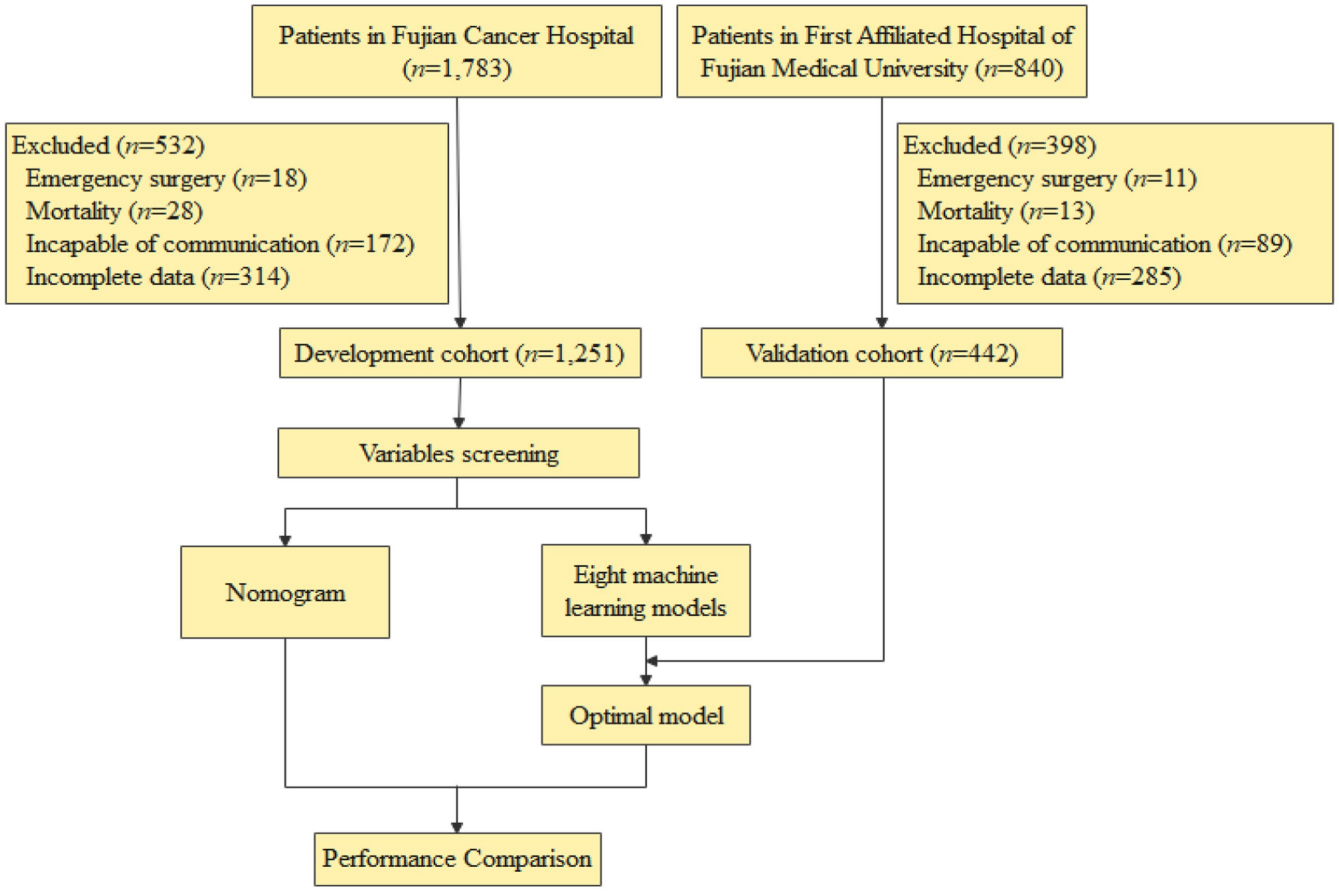
Flowchart depicting patient allocation in both the development and validation cohorts.

Exclusion Criteria: patients were excluded if they (1) required emergency surgery (e.g., obstruction, bleeding); (2) died within 30 days postoperatively; (3) had communication barriers (e.g., language barriers, severe cognitive impairment); (4) lacked complete hospitalization or 1-month follow-up data. The study flowchart is presented in Figure 1.

### 2.2 Definition

At our institution, esophageal cancer patients routinely undergo follow-up appointments 1 month after surgery. The GLIM criteria were employed to diagnose malnutrition during these follow-ups, with assessments conducted by trained research assistants or nurses. This method is recognized for its reliability in evaluating the nutritional status of cancer patients (29–31). The GLIM criteria involve a two-step diagnostic process: initial screening using the Nutritional Risk Screening 2002 (NRS-2002) tool to identify at-risk individuals (scores ≥3 indicating malnutrition risk) (32), followed by confirmation and severity assessment based on phenotypic and etiological criteria. Specifically, malnutrition diagnosis requires at least one phenotypic criterion (e.g., weight loss, low BMI, or reduced muscle mass) and one etiological criterion (e.g., decreased food intake/assimilation, inflammation, or disease burden) (33, 34). Given the chronic inflammatory nature of esophageal cancer, all patients in this study were considered to meet the etiological criteria related to disease burden and inflammation.

According to the diagnostic criteria established by the Asian Working Group for Sarcopenia (AWGS), sarcopenia is characterized by the presence of low skeletal muscle mass combined with decreased muscle strength and/or impaired physical performance (35). In this study, preoperative computed tomography (CT) images at the third lumbar vertebra (L3) level were analyzed using SliceOmatic software (v5.0, TomoVision) to quantify skeletal muscle area (SMA, cm^2^; Supplementary Figure 1). The skeletal muscle index (SMI, cm^2^/m^2^) was subsequently calculated by normalizing SMA to height squared. Sex-specific thresholds for low SMI were applied based on AWGS recommendations: 34.9 cm^2^/m^2^ for females and 40.8 cm^2^/m^2^ for males (36). Muscle strength assessment was performed using standardized handgrip dynamometry, with diagnostic cutoffs defined as <28 kg for males and <18 kg for females (35).

Smoking status was categorized into current smokers and non-smokers (including former and never smokers) (37). Drinking was defined as consuming at least one alcoholic drink per week (38). Postoperative complications were evaluated using the Clavien-Dindo classification, with major complications defined as those of grade IIIa or higher (39). Chronic pulmonary diseases, as considered in this study, encompass conditions like chronic obstructive pulmonary disease (COPD), asthma, restrictive lung disease, and obstructive sleep apnea (40). Cerebrovascular diseases encompassed intracerebral hemorrhage, ischemic stroke, and transient ischemic attack (TIA) (41). Ischemic heart disease was defined as impaired blood flow to the myocardium, encompassing acute myocardial infarction, stable angina, and chronic ischemic heart disease, potentially leading to heart failure (42).

### 2.3 Statistical analysis

Data conforming to a normal distribution were presented as mean ± standard deviation (SD) and analyzed using an independent-samples *t*-test. Non-normally distributed data were expressed as medians (first quartile [Q1] and third quartile [Q3]) and analyzed using the Wilcoxon rank-sum test. Categorical variables were described using frequencies and percentages, and compared using the Chi-square test or Fisher’s exact test, as appropriate. Feature selection was performed using the least absolute shrinkage and selection operator (LASSO) logistic regression method. Eight machine learning algorithms were employed for model development: Random Forest (RF), K-Nearest Neighbors (KNN), Gaussian Naive Bayes (GNB), Partial Least Squares (PLS), Neural Network (NN), TreeBagger (TB), Extreme Gradient Boosting (XGBoost), and Support Vector Machine (SVM). The SHapley Additive exPlanations (SHAP) framework was applied to interpret feature contributions in the top-performing model. Multivariate logistic regression analysis of LASSO-identified predictors facilitated the identification of independent risk factors for malnutrition. A dynamic nomogram was then developed based on these independent risk factors. Model discrimination was assessed by calculating the area under the curve (AUC) of the receiver operating characteristic (ROC) curve, while calibration was evaluated using calibration plots along with the Hosmer–Lemeshow (H-L) test. Decision curve analysis (DCA) was utilized to estimate the model’s clinical utility. Statistical significance was established at a two-tailed *P* of <0.05.

## 3 Results

### 3.1 Patient characteristics

Among the 1,693 patients who underwent esophageal cancer surgery, 46.8% (792/1,693) developed postoperative malnutrition. The incidence of malnutrition was 45.4% (568/1,251) in the development cohort and 50.7% (224/442) in the validation cohort. Comparative analysis of baseline characteristics revealed no significant differences between the two cohorts, except for education level and hypertension (Supplementary Table 1), indicating comparability across most examined parameters.

### 3.2 Machine learning model construction

**Figure 2:**
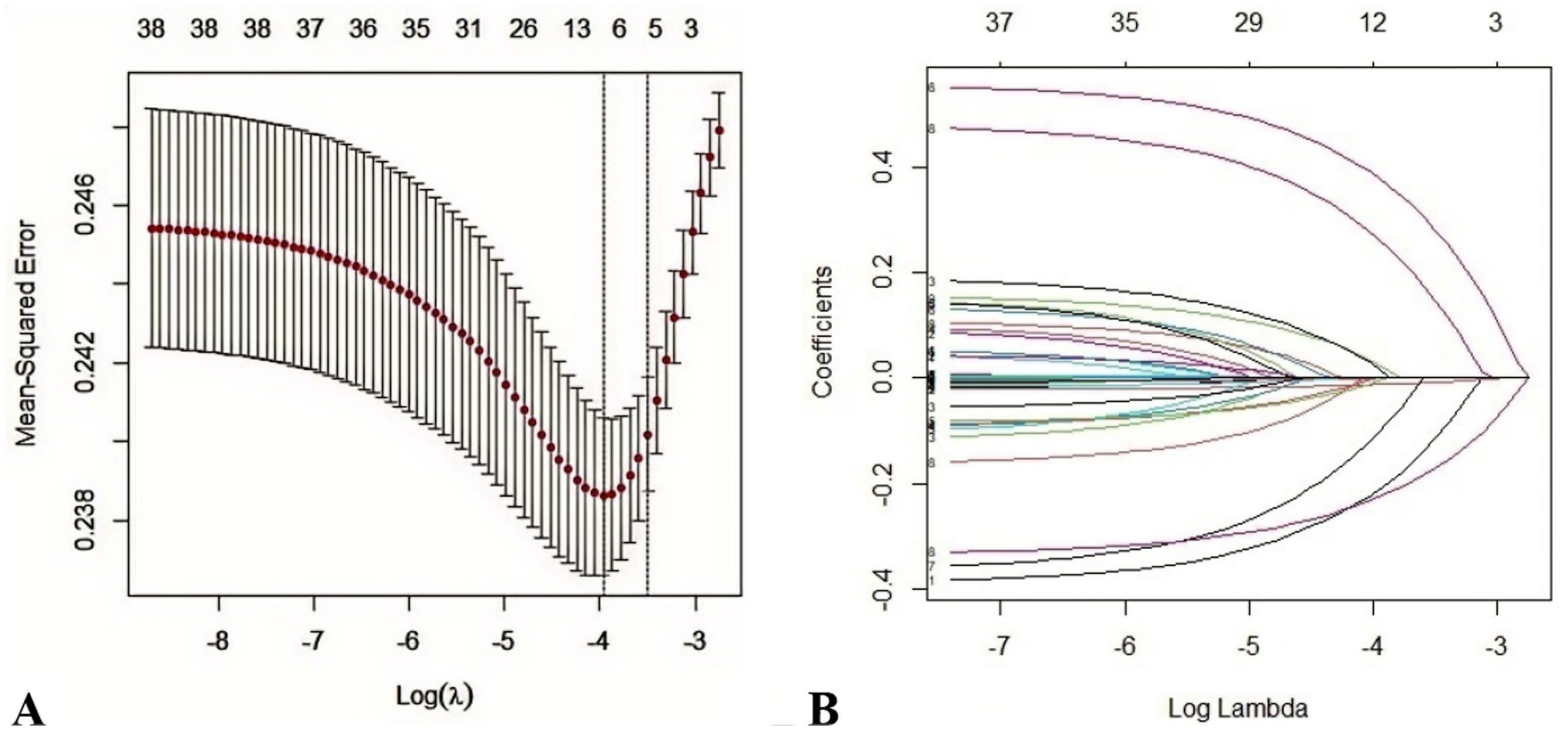
Feature selection via the LASSO regression model. **(A)** Selection of the optimal penalty parameter (*λ*) using 10-fold cross-validation based on the minimum criterion. The vertical black line indicates the optimal *λ* = 0.019, balancing model complexity and accuracy. **(B)** LASSO coefficient profiles showing eight optimal predictors identified at the selected log(λ) value.

In the development cohort, the LASSO regression, optimized via 10-fold cross-validation (*λ* = 0.019), identified eight predictors of postoperative malnutrition: female sex, age, preoperative body mass index (BMI) < 18.5 kg/m^2^, ASA score III–IV, drinking, diabetes mellitus, neoadjuvant therapy, and preoperative sarcopenia (Figure 2). This method minimizes overfitting while prioritizing variables with robust clinical relevance.

Based on these selected variables, eight machine learning models were constructed and evaluated: RF, KNN, GNB, PLS, NN, TB, XGBoost, and SVM. Comprehensive model performance was evaluated using sensitivity, specificity, accuracy, positive predictive value (PPV), negative predictive value (NPV), precision, recall, F1-score, Youden’s index, and AUC.

**Figure 3:**
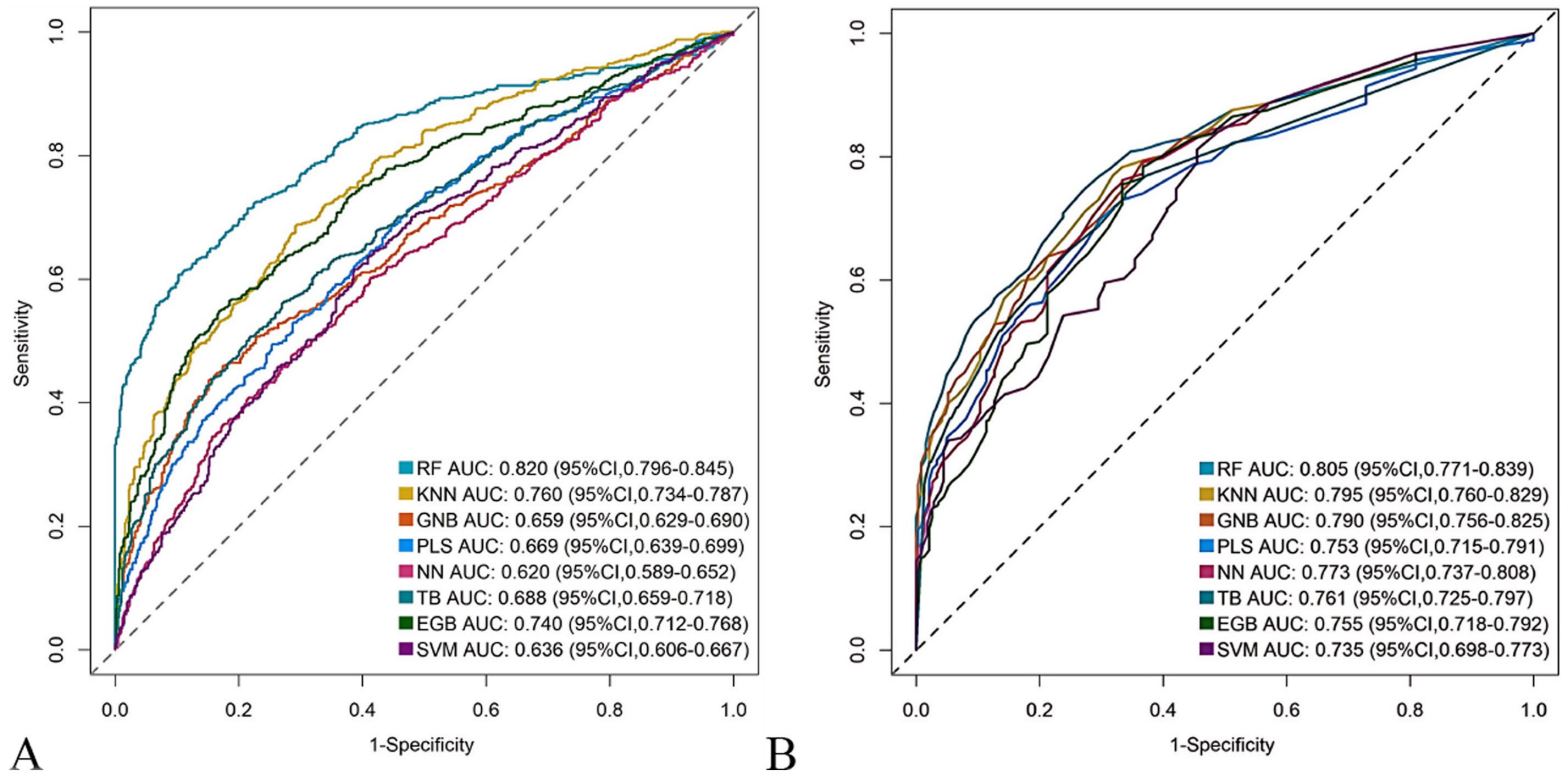
Receiver operating characteristic (ROC) curves of the eight machine learning models. **(A)** Development cohort. **(B)** Validation cohort.

**Figure 4:**
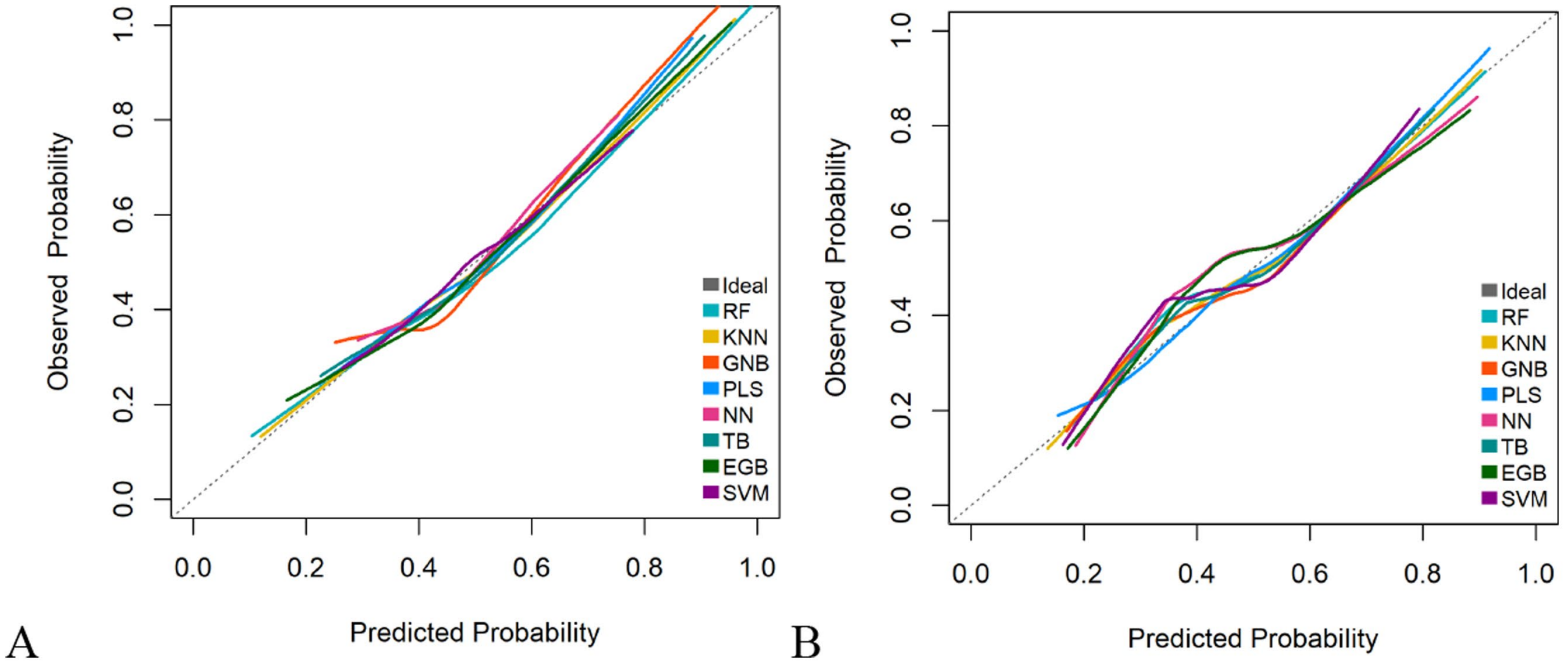
Calibration plots of the eight machine learning models. **(A)** Development cohort. **(B)** Validation cohort.

**Figure 5:**
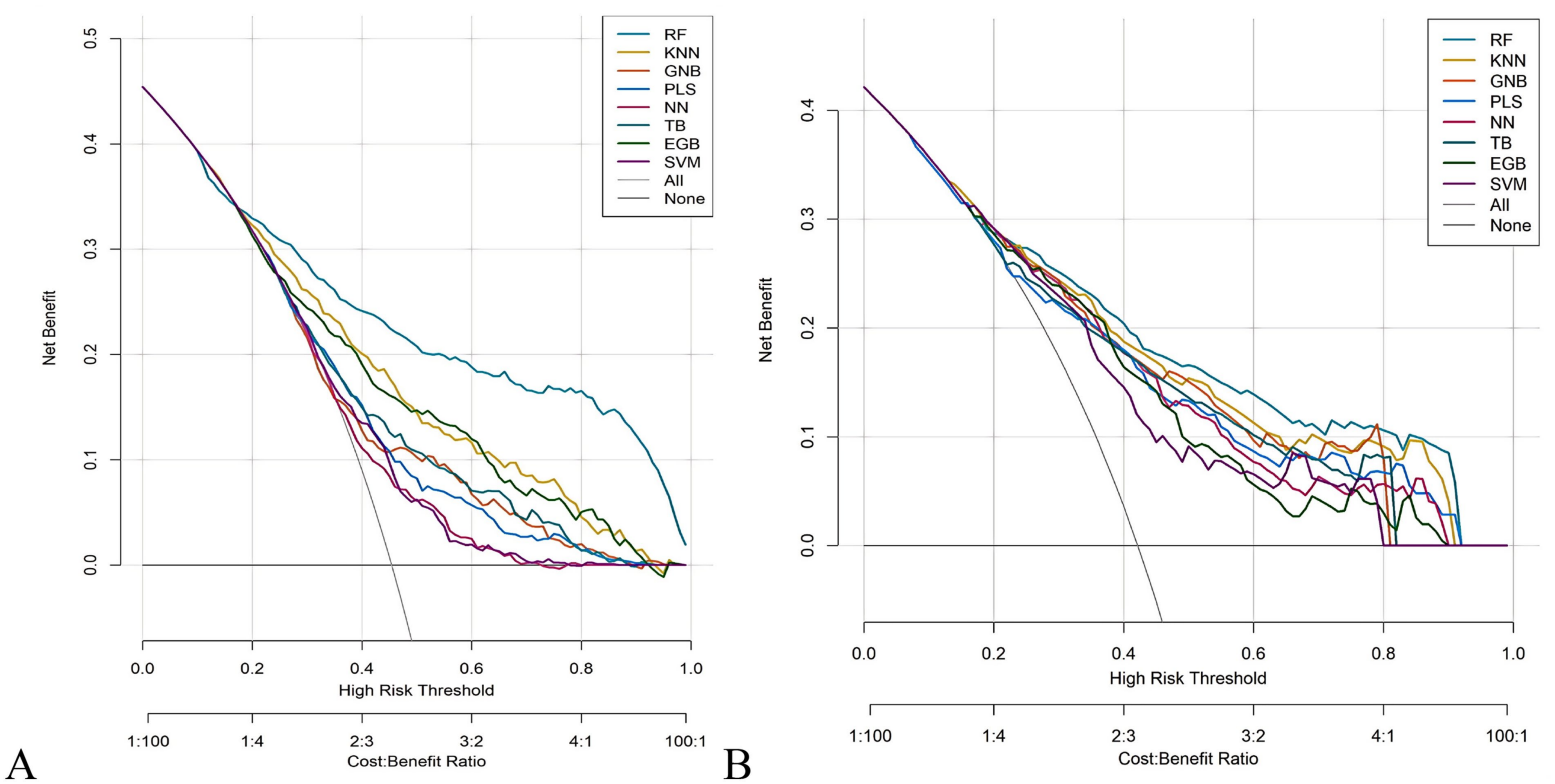
Decision curve analysis (DCA) of the eight machine learning models. **(A)** Development cohort. **(B)** Validation cohort.

The RF model demonstrated optimal overall performance in both development and validation cohorts. In the development cohort, RF achieved the highest scores in discriminative ability (AUC = 0.820, 95% CI: 0.796–0.845), accuracy (0.766), NPV (0.733), Youden’s index (0.505), and F1-score (0.702). In the validation cohort, RF maintained superiority in specificity (0.724), accuracy (0.735), PPV (0.665), precision (0.665), Youden’s index (0.475), F1-score (0.706), achieving an AUC of 0.805 (95% CI: 0.771–0.839) (Figure 3 and Supplementary Figures 2, 3). The RF model demonstrated excellent calibration accuracy in both cohorts (Figure 4), with DCA further validating its clinical utility. Superior net benefits were observed across threshold probabilities of 15–100% (development) and 13–92% (validation) (Figure 5), highlighting its advantages over alternative strategies within clinically relevant risk ranges.

### 3.3 Model interpretability

**Figure 6:**
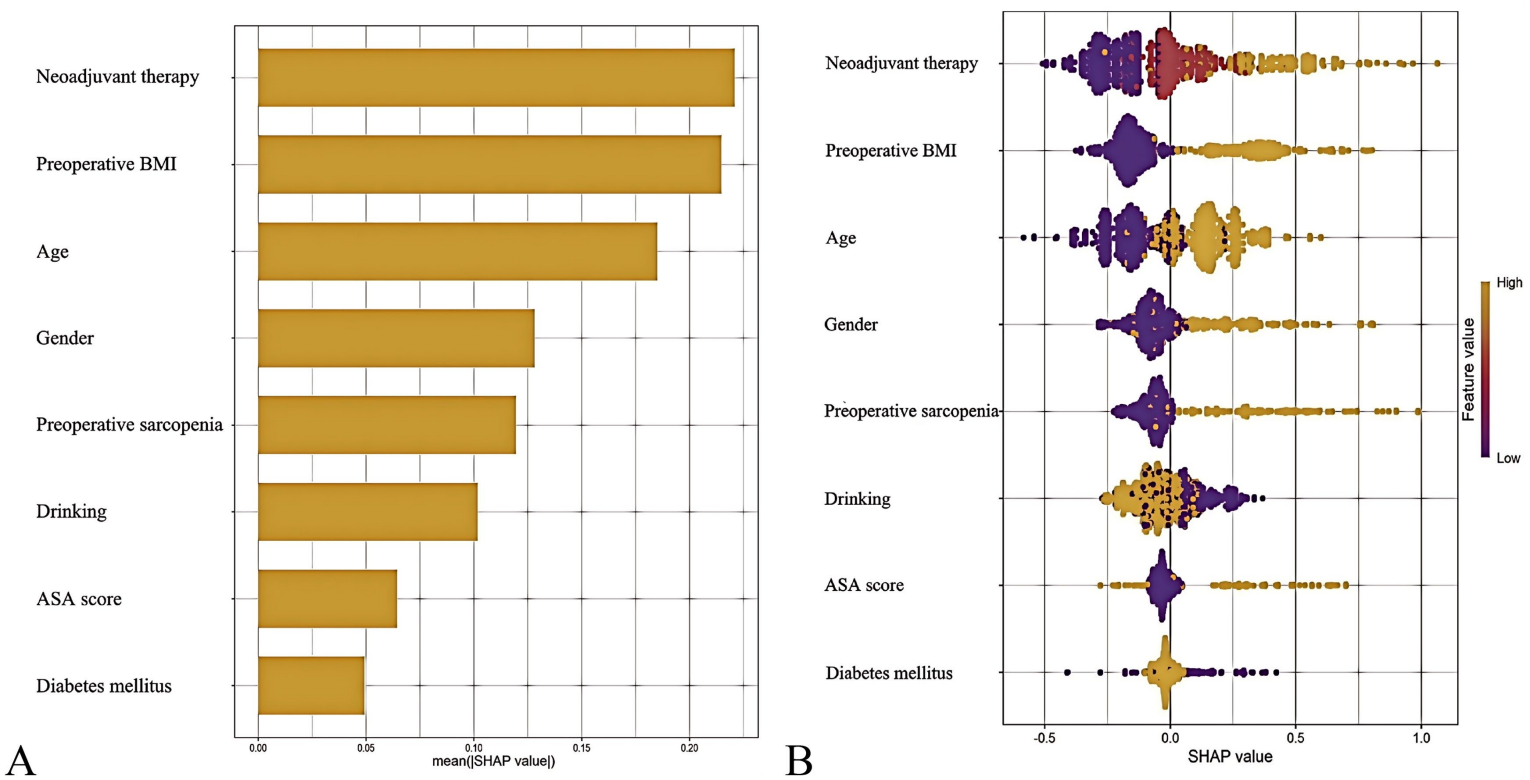
SHapley Additive exPlanations (SHAP) analysis of the predictive model. **(A)** Mean absolute SHAP values for top predictors (bar plot). **(B)** Individual prediction explanation (waterfall plot).

The out-of-bag (OOB) error rate decreased with increasing numbers of decision trees and stabilized after 300 trees, indicating optimal performance of the random forest model (Supplementary Figure 4). To quantify variable contributions to postoperative malnutrition risk, the SHAP framework was applied. As shown in the SHAP summary plot (Figure 6), the top predictors of postoperative malnutrition, ranked by mean absolute SHAP values, were: neoadjuvant therapy (SHAP value: 0.24), preoperative BMI (0.23), age (0.18), female sex (0.15), and preoperative sarcopenia (0.12).

### 3.4 Construction of the nomogram in the development cohort

**Figure 7:**
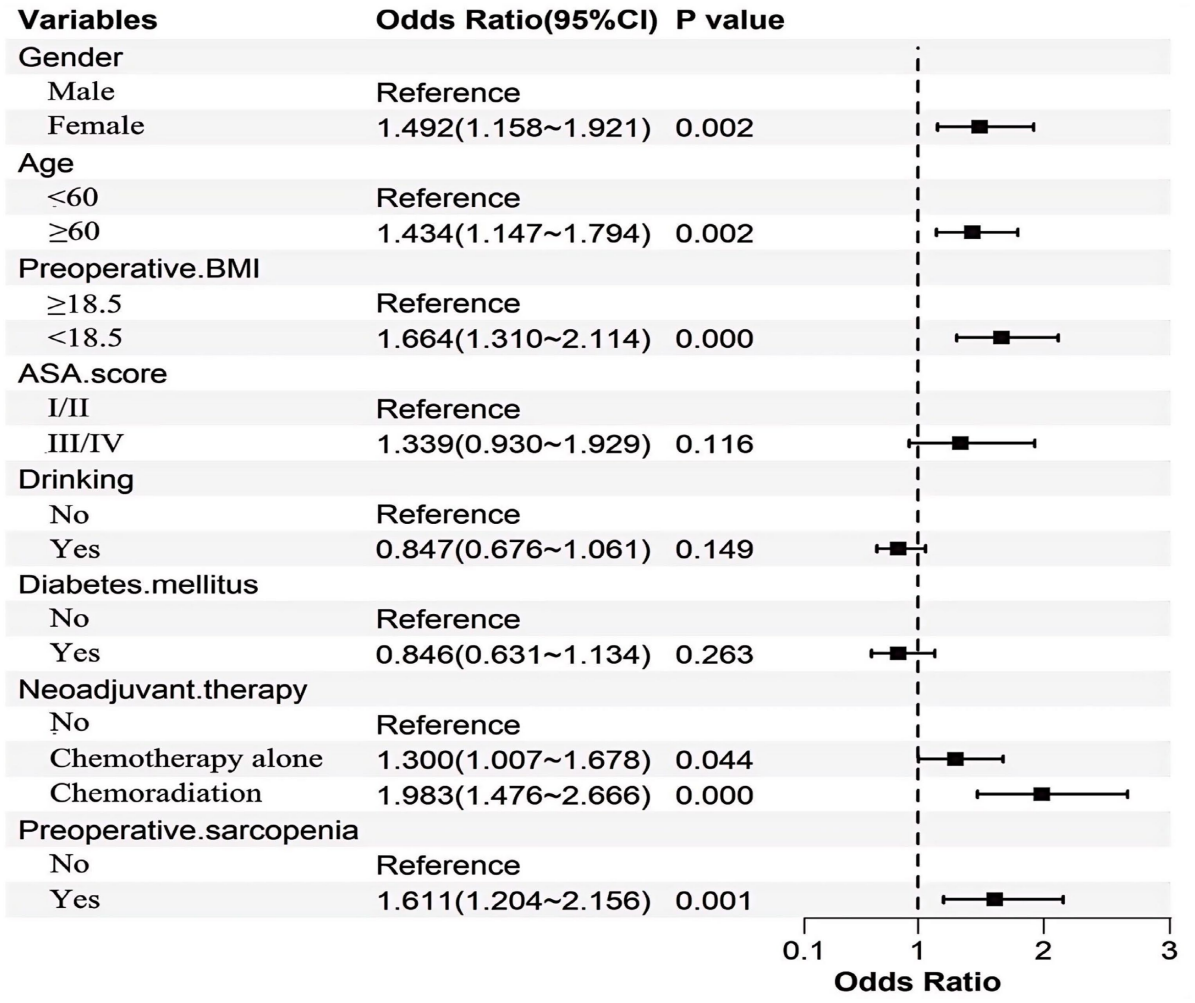
Forest plot of multivariable logistic regression.

To enhance clinical utility, continuous variables were dichotomized based on optimal cutoff values determined by ROC analysis. The optimal cutoff for age was 60 years, with an AUC of 0.662 (95% CI: 0.632–0.693), as detailed in Supplementary Figure 5. Subsequent multivariate logistic regression analysis identified female sex, age ≥ 60 years, BMI < 18.5 kg/m^2^, neoadjuvant therapy, and preoperative sarcopenia. The statistical significance and effect sizes of these predictors are illustrated in Figure 7.

**Figure 8:**
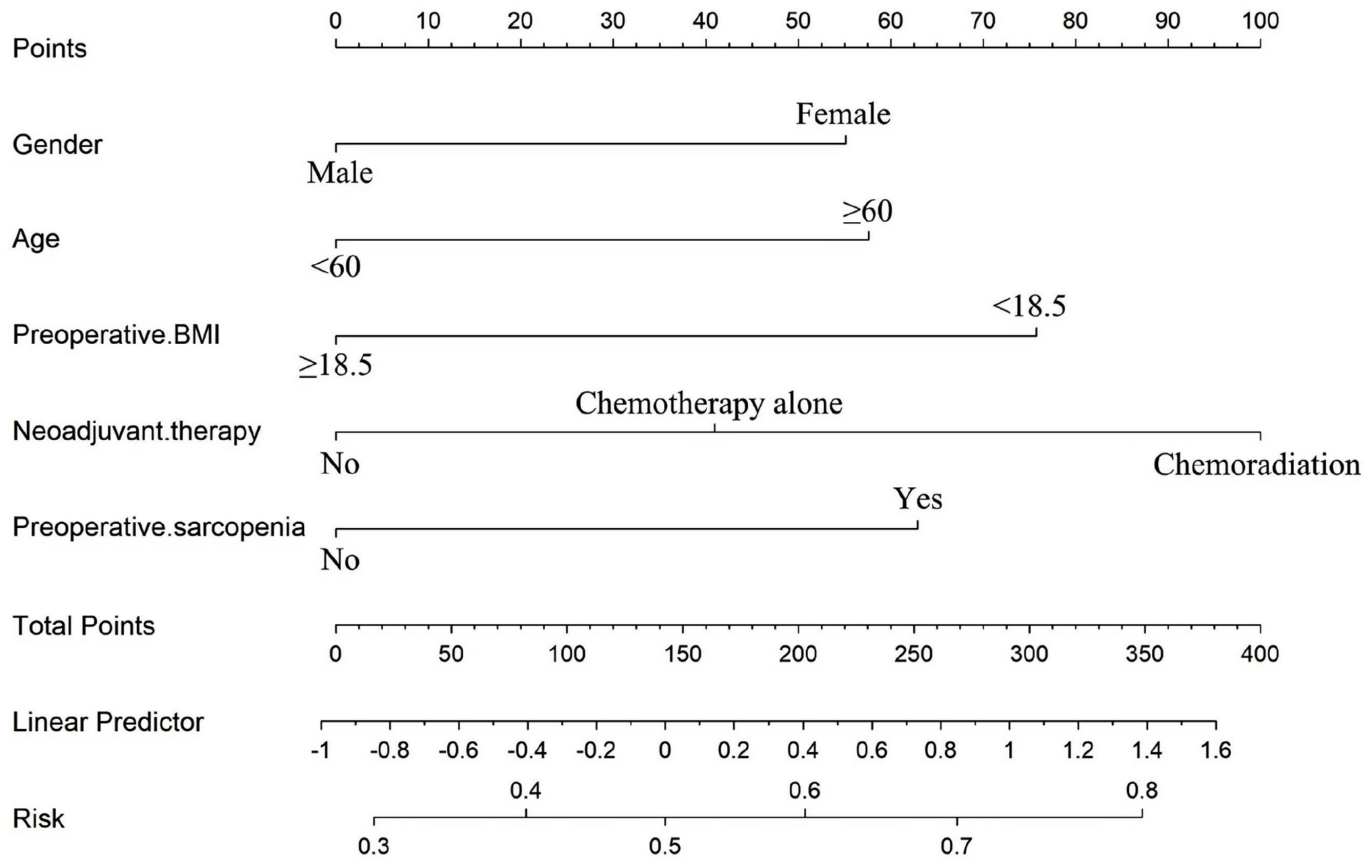
Nomogram for the individualized prediction of malnutrition after esophageal cancer surgery.

**Figure 9:**
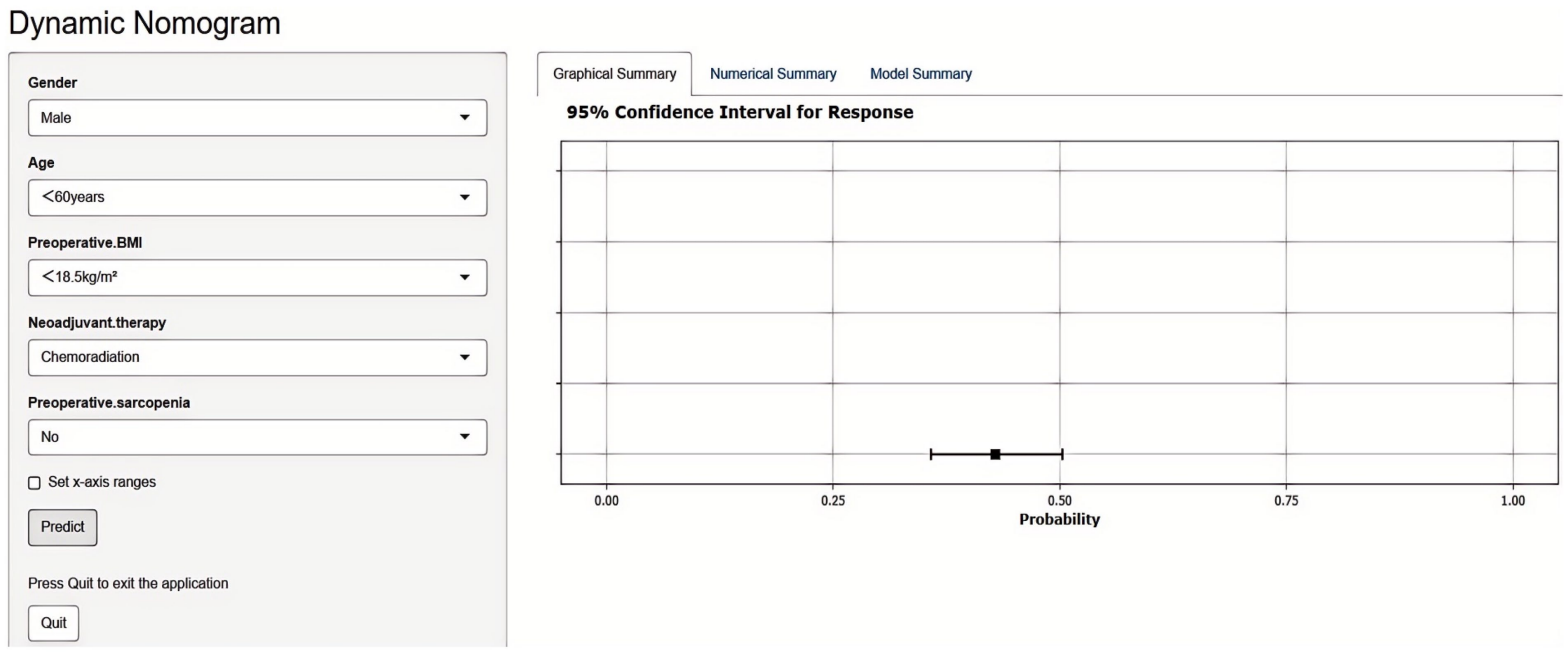
Dynamic nomogram for predicting postoperative malnutrition in esophageal cancer patients. Upon entering the relevant features in the left panel, the predicted probability of malnutrition is displayed in the right panel.

Based on these independent predictors, a nomogram was developed to estimate the likelihood of postoperative malnutrition (Figure 8). To further improve clinical applicability, an interactive online version of the nomogram was created (Figure 9) and is publicly available at: https://lzmdoc123456789.shinyapps.io/pomnt/.

### 3.5 Validation of the nomogram

**Figure 10:**
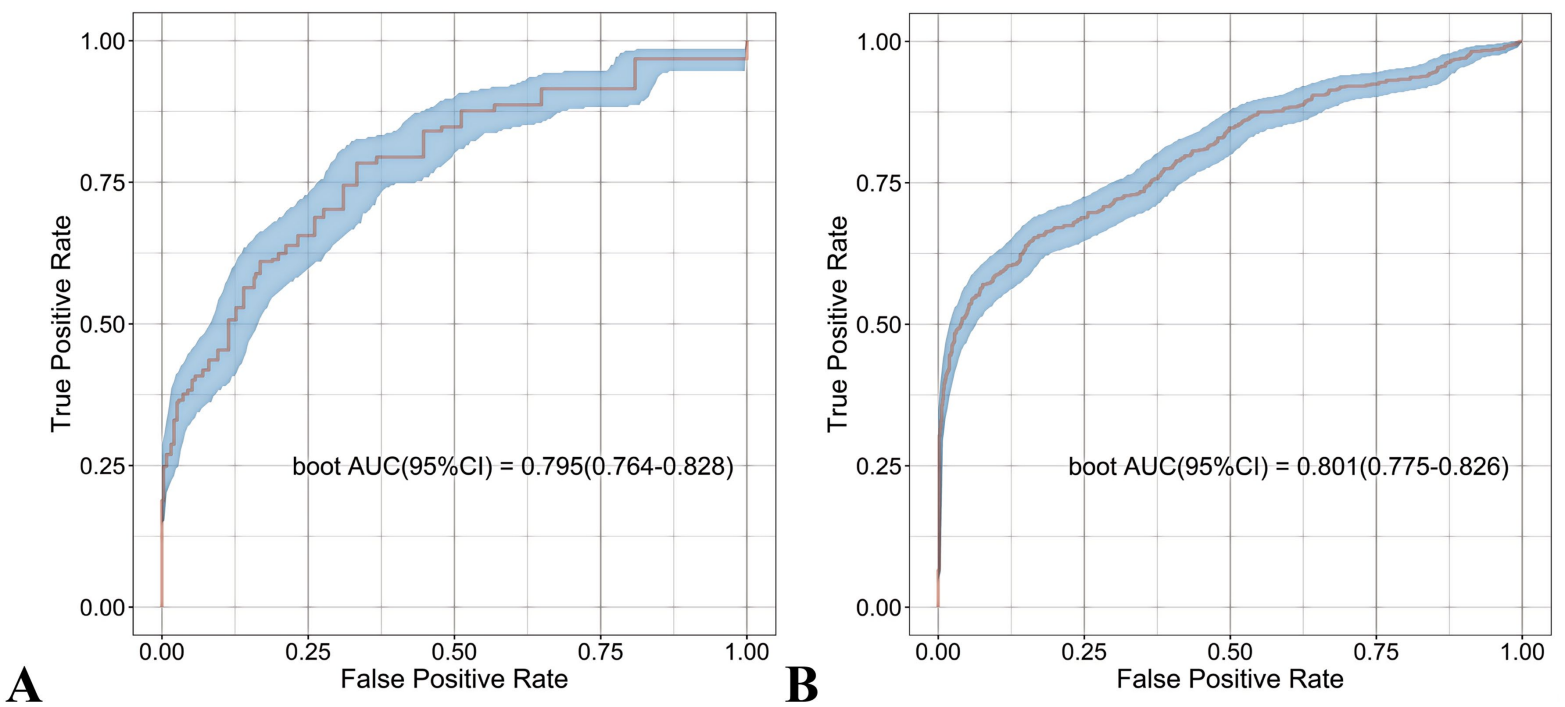
Receiver operating characteristic (ROC) curves of the nomogram. **(A)** Development cohort. **(B)** Validation cohort.

The nomogram demonstrated robust predictive accuracy for postoperative malnutrition, with AUC values of 0.801 (95% CI: 0.775–0.826) in the development cohort and 0.795 (95% CI: 0.764–0.828) in the validation cohort (Figure 10). Notably, no statistically significant difference in AUC was observed between the nomogram and the top-performing machine learning model, RF (Table 1).

**Table 1:** Comparison of AUC values between the RF model and nomogram.

Model Type	AUC (95% CI)	*P*
Development cohort		0.231
RF	0.820 (95% CI: 0.796–0.845)	
Nomogram	0.801 (95% CI: 0.775–0.826)	
Validation cohort		0.080
RF	0.805 (95% CI: 0.771–0.839)	
Nomogram	0.795 (95% CI: 0.761–0.830)	

**Figure 11:**
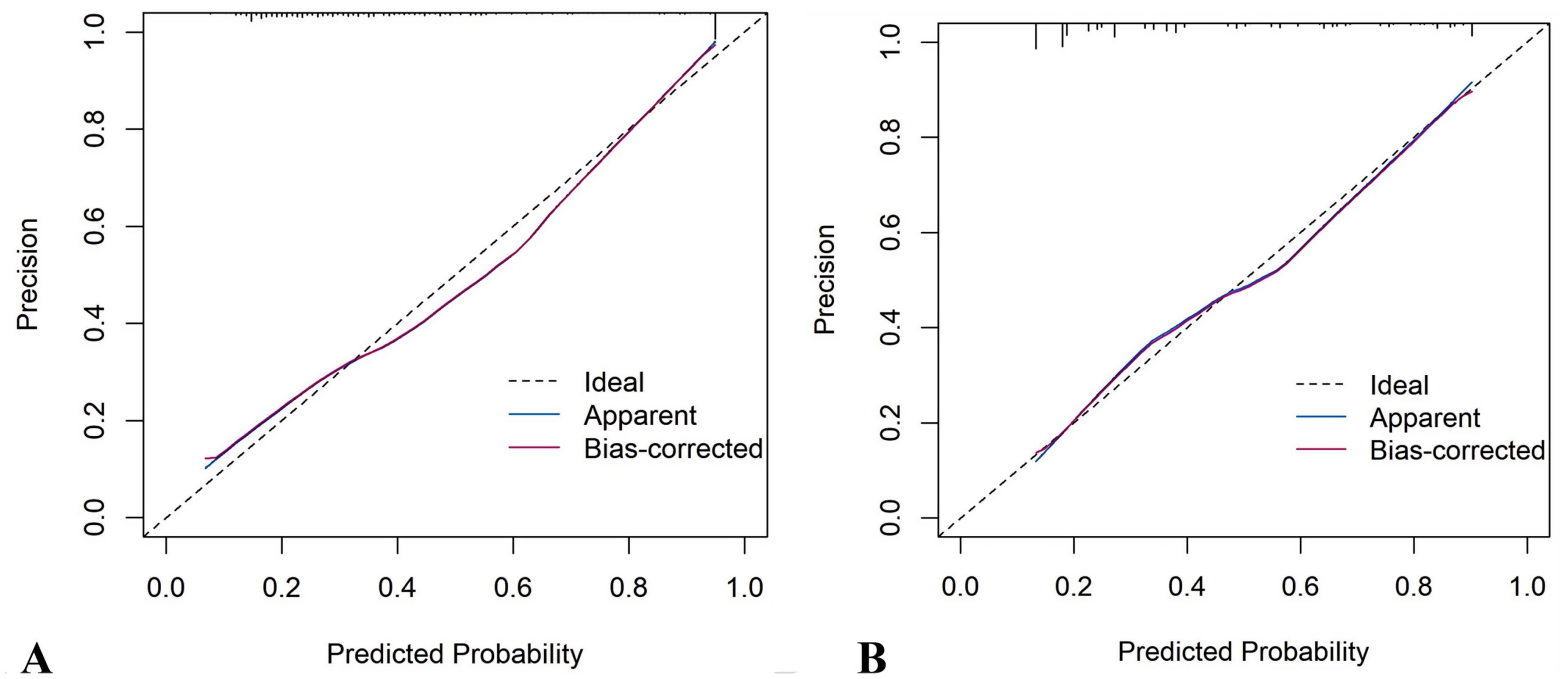
Calibration plots of the nomogram. **(A)** Development cohort. **(B)** Validation cohort.

**Figure 12:**
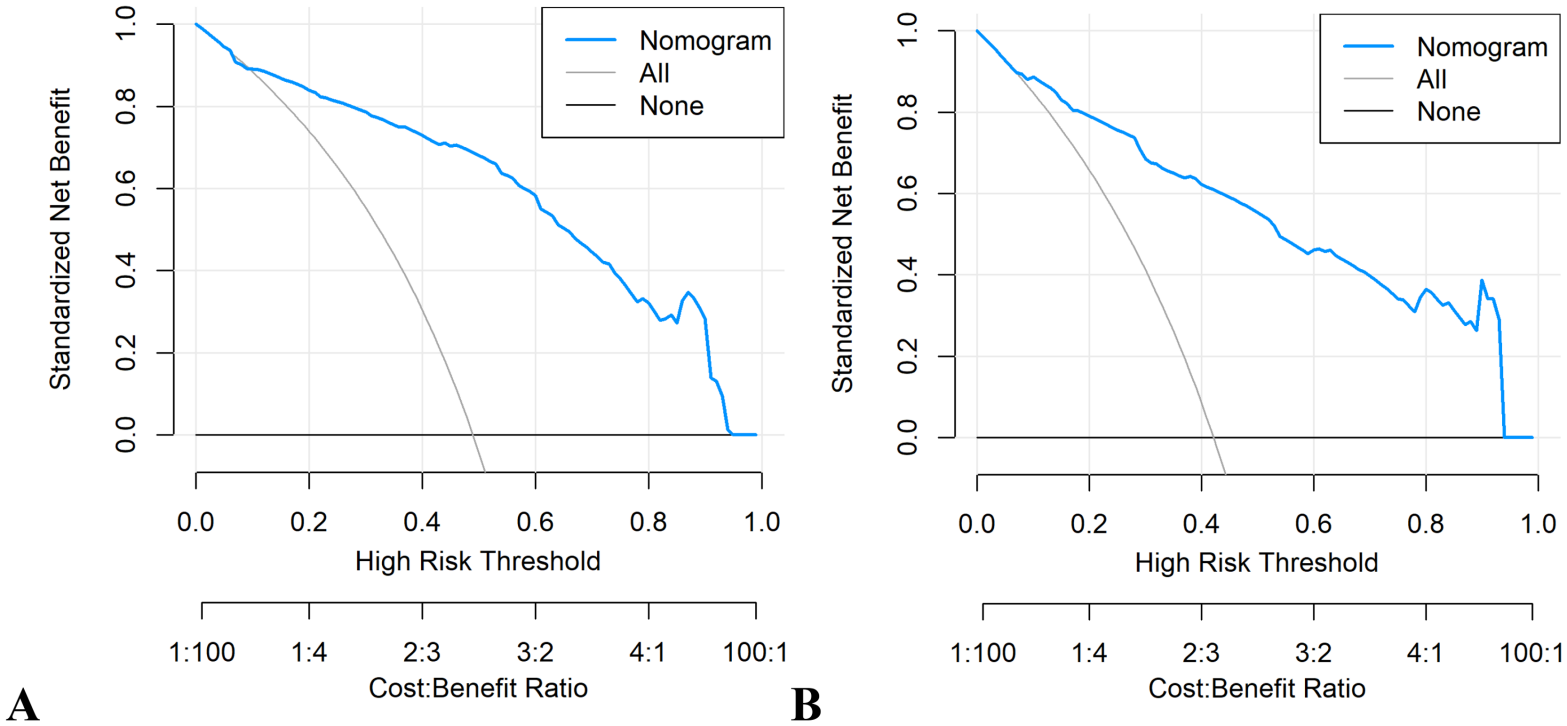
Decision curve analysis (DCA) plots of the nomogram. **(A)** Development cohort. **(B)** Validation cohort.

It demonstrated good calibration in both cohorts, with calibration curves showing close agreement between predicted and observed probabilities (Figure 11). The H-L test results were non-significant (development: *χ*^2^ = 6.99, *p* = 0.635; validation: *χ*^2^ = 7.18, *p* = 0.620). DCA confirmed clinical utility, showing superior net benefits across threshold probabilities of 6–94% (development) and 8–95% (validation) compared to “treat all” or “treat none” strategies (Figure 12 and Supplementary Figure 6).

## 4 Discussion

### 4.1 Epidemiological context

In contemporary clinical practice, advancements in multimodal therapeutic approaches and surgical techniques have significantly improved survival outcomes for esophageal cancer patients, particularly those eligible for curative interventions (43, 44). As the survival rate for patients increases, it becomes essential to focus on factors beyond just cancer-related outcomes in their long-term care, particularly the importance of maintaining good nutritional status. However, the prevalence of malnutrition varies significantly across studies due to differences in diagnostic criteria and follow-up duration. For instance, Schandl et al. (45) prospectively analyzed 351 esophagectomy patients and reported that 35.6% (125/351) experienced significant postoperative weight loss. In contrast, Martin et al. (46) observed a consistently high proportion of patients facing malnutrition risk: 77% prior to treatment, 71% at 2 months post-treatment, 85% at 4 months, and 72% at 6 months. Lidoriki et al. (47) found that patients undergoing esophagectomy experienced the most significant postoperative weight loss, with a mean reduction of 16.2 ± 9.6% at 6 months. Notably, our findings provide enhanced validity through rigorous application of GLIM criteria and a large multicenter cohort (*n* = 1,693), ensuring standardized diagnosis and generalizability of the observed 46.8% malnutrition incidence.

### 4.2 Predictive models

Given the high prevalence and profound clinical consequences of postoperative malnutrition in esophageal cancer patients, early identification of high-risk individuals and optimized nutritional support are critical for preserving lean body mass and metabolic reserve (48–50). The nomogram, as a visual predictive tool, translates complex regression models into intuitive graphical interfaces, significantly enhancing clinical accessibility compared to traditional scoring systems (51). This study developed and validated a robust nomogram demonstrating strong discriminative ability (AUC = 0.795–0.801) and calibration in both internal and external cohorts. DCA confirmed its clinical utility across threshold probabilities of 6–95%, supporting its role in guiding interventions like prophylactic feeding jejunostomy for high-risk patients.

To advance predictive performance, we explored machine learning algorithms capable of capturing complex variable interactions. Among eight ML models, the RF demonstrated optimal performance (AUC = 0.805–0.820). Notably, The RF model showed marginally higher discriminative ability than the nomogram, though no significant difference was observed. Although machine learning models may offer slight accuracy gains, their “black-box” nature and computational demands limit real-world implementation, particularly in resource-limited settings (52). Our findings highlight that the nomogram’s balance of accuracy, interpretability, and simplicity better aligns with clinical pragmatism.

### 4.3 Key risk factors

Our findings identified female sex as an independent predictor of postoperative malnutrition. Compared to males, females generally exhibit a higher proportion of adipose tissue and lower skeletal muscle mass. Following surgery, the increased demand for nutrients—particularly protein, required for tissue repair and muscle preservation—may disproportionately affect females, as reduced skeletal muscle mass limits metabolic reserves and adaptive capacity, thereby heightening the risk of malnutrition (24, 53). Furthermore, female patients often face heightened psychological stressors, such as anxiety and depression, as well as socioeconomic challenges including caregiving responsibilities and financial constraints. These factors may compromise dietary adherence and exacerbate nutritional deficits, potentially establishing a bidirectional pathway that contributes to the progression of malnutrition (54, 55).

Advanced age was significantly associated with malnutrition risk, likely due to multifactorial physiological and social determinants. Key contributors include age-related sarcopenia, which diminishes muscle mass and metabolic reserve, and polypharmacy that may interfere with nutrient absorption or appetite regulation. Chronic comorbidities such as diabetes or cardiovascular diseases further exacerbate metabolic dysregulation and dietary restrictions (56). Reduced mobility and functional decline limit access to nutrient-dense foods, while diminished gustatory and olfactory senses lower food enjoyment and intake (57). Prolonged postoperative recovery in elderly patients often necessitates increased protein and caloric demands, yet diminished gastrointestinal efficiency and psychological stressors (e.g., depression, social isolation) create barriers to meeting these needs (58, 59). These intersecting factors highlight the importance of tailored nutritional interventions and comprehensive geriatric assessments in mitigating malnutrition risk in older surgical populations.

According to the Malnutrition Universal Screening Tool (MUST) and the European Society of Clinical Nutrition and Metabolism (ESPEN) Malnutrition Diagnostic Criteria, a BMI of less than 18.5 kg/m^2^ is recognized as indicative of malnutrition (60, 61). This threshold is also consistent with the underweight definition provided by the World Health Organization (WHO) and is one of the indicators in the GLIM criteria (31, 62). Therefore, we used 18.5 kg/m^2^ as the cutoff for BMI in our study. Preoperative low BMI was identified as an independent risk factor for postoperative malnutrition in our study, consistent with findings from previous studies (24, 25). As a type of upper gastrointestinal malignancy, esophageal cancer directly impacts food intake. The majority of esophageal cancer cases are diagnosed at an advanced stage, characterized by progressive dysphagia and significant weight loss as the predominant symptomatic manifestations. The intrinsic characteristics of esophageal cancer predispose patients to a higher likelihood of experiencing preoperative low BMI (5, 7). Surgical interventions, especially major operations like esophagectomy, significantly stress the body and elevate the requirements for energy and protein. A low BMI preoperatively indicates insufficient nutritional reserves, making it difficult for these patients to meet the increased demands for energy and nutrients following surgery.

The efficacy of preoperative oncological treatments (e.g., chemotherapy or chemoradiotherapy) in esophageal cancer management is well-established (63, 64). However, our study revealed a strong association between neoadjuvant therapy and postoperative malnutrition. This correlation may be attributed to treatment-related toxicities, including radiation-induced pneumonitis, esophagitis, dysphagia, esophageal strictures, and reduced physical activity, all of which can directly impair nutritional intake or exacerbate catabolic states (65). Prolonged inflammation from chemoradiotherapy may further disrupt energy metabolism by upregulating pro-inflammatory cytokines (e.g., TNF-*α*, IL-6), accelerating muscle proteolysis and adipose tissue breakdown (66). Additionally, chemotherapy-induced gastrointestinal mucositis and alterations in gut microbiota composition can compromise nutrient absorption and utilization (67, 68). These physiological insults are compounded by treatment-related anorexia and taste alterations, which diminish dietary adherence and caloric intake.

Preoperative sarcopenia was identified as an independent risk factor for postoperative malnutrition in this study. Patients with sarcopenia exhibit significantly reduced skeletal muscle mass and diminished protein reserves, rendering them vulnerable to accelerated protein catabolism under postoperative traumatic stress. Furthermore, the chronic systemic inflammatory status associated with sarcopenia is exacerbated by surgical trauma, leading to enhanced muscle proteolysis and protein depletion. Additionally, compromised physical endurance in sarcopenic patients restricts early postoperative ambulation, which may adversely affect appetite regulation, gastrointestinal function, and subsequent nutritional intake. This functional decline creates a cyclical relationship, where reduced mobility further accelerates muscle loss and impairs metabolic homeostasis (69–71).

### 4.4 Limitations

Nonetheless, this study is subject to certain limitations. Firstly, the development and validation cohorts were independently conducted at separate single-center institutions within the same city. Hence, the applicability of our findings may not be generalizable, particularly for patients in Western countries, due to variations in factors such as tumor histology and the location of the primary tumor (72). Secondly, the study did not account for various clinical aspects such as socioeconomic status, dietary patterns, eating behaviors, and psychological well-being. Thirdly, we only used the GLIM criteria to assess malnutrition and did not compare the results with other assessment tools such as the Subjective Global Assessment (SGA). Finally, nutritional status was evaluated 1 month post-surgery without any subsequent follow-up.

## 5 Conclusion

This study presents a dual approach to predict postoperative malnutrition in esophageal cancer patients: a high-accuracy RF model and a clinically interpretable nomogram. While the RF model offers marginally superior predictive performance, the nomogram prioritizes usability through visual risk stratification, making it ideal for integration into clinical workflows (e.g., preoperative counseling) and electronic health records (EHRs). By embedding these tools into routine practice, clinicians can proactively tailor nutritional interventions—such as prophylactic jejunostomy placement for high-risk patients or transient nasojejunal feeding for low-risk individuals—while enhancing patient-clinician communication through intuitive risk visualization.

## Data Availability

The original contributions presented in the study are included in the article/Supplementary material, further inquiries can be directed to the corresponding authors.
