# Supplementary material for: Machine learning and the nomogram as the accurate tools for predicting postoperative malnutrition risk in esophageal cancer patients
Source: Front Nutr. 2025 Jun 18;12:1606470. doi: 10.3389/fnut.2025.1606470 (PMC12214435; doi:10.3389/fnut.2025.1606470)
Supplement: Supplementary file 1 [file Data_Sheet_1.pdf]

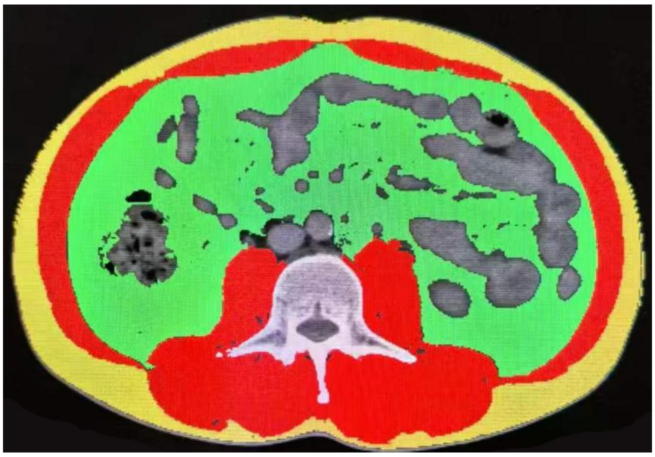

SUPPLEMENTARY FIGURE 1
Skeletal muscle area at the level of the third lumbar vertebra on CT imaging.

SUPPLEMENTARY TABLE 1 Clinicopathological features of patients in the development and validation cohorts.

**Features**	**Development cohort (n=1,251)**	**Validation cohort (n=442)**	$x^2/t/U$	***P* value**
Gender			1.246	0.264
Male	923 (73.8%)	314 (71.0%)		
Female	328 (26.2%)	128 (29.0%)		
Age (years), mean (SD)	60.7±11.8	61.5±12.1	1.265	0.206
Education level			6.469	0.011
Senior high school and above	167 (13.3%)	81 (18.3%)		
Junior middle school and below	1,084 (86.7%)	361 (81.7%)		
Marital status			0.504	0.478
Spouse	869 (69.5%)	299 (67.6%)		
No spouse	382 (30.5%)	143 (32.4%)		
Family history			1.042	0.307
Yes	159 (12.7%)	48 (10.9%)		
No	1,092 (87.3%)	394 (89.1%)		
Preoperative BMI, kg/m^2			1.795	0.180
<18.5	382 (30.5%)	120 (27.1%)		
≥18.5	869 (69.5%)	322 (72.9%)		
ASA score			0.236	0.627
I/II	1,122 (89.7%)	400 (90.5%)		
III/IV	129 (10.3%)	42 (9.5%)		
Smoking			1.203	0.273
Yes	630 (50.4%)	236 (53.4%)		
No	621 (49.6%)	206 (46.6%)		

Drinking			1.796	0.180
Yes	523 (41.8%)	201 (45.5%)		
No	728 (58.2%)	241 (54.5%)		
FEV_1/FVC, % , mean (SD)	87.9±4.6	87.6±4.8	1.338	0.181
History				
Hypertension	303 (24.2%)	130 (29.4%)	4.624	0.032
Hyperlipidemia	260 (20.8%)	106 (24.0%)	1.972	0.160
Diabetes mellitus	217 (17.3%)	68 (15.4%)	0.898	0.343
Chronic pulmonary disease	170 (13.6%)	70 (15.8%)	1.357	0.244
Cerebrovascular disease	149 (11.9%)	54 (12.2%)	0.029	0.865
Ischemic heart disease	151 (12.1%)	59 (13.3%)	0.491	0.483
Previous thoracic or abdominal surgery	199 (15.9%)	77 (17.4%)	0.548	0.459
Neoadjuvant therapy			3.903	0.142
Chemotherapy alone	414 (33.1%)	150 (33.9%)		
Chemoradiation	263 (21.0%)	110 (24.9%)		
No	574 (45.9%)	182 (41.2%)		
Preoperative sarcopenia			0.946	0.331
Yes	223 (17.8%)	88 (19.9%)		
No	1,028 (82.2%)	354 (80.1%)		
Preoperative blood test				
WBC, $\times 10^9$/L, median (Q1, Q3)	6.6 (5.4, 7.6)	6.7 (5.5, 7.8)	1.502	0.133
Platelets, $\times 10^9$/L, median (Q1, Q3)	242 (198, 297)	244 (206, 301)	1.187	0.235
Hemoglobin, g/L, median (Q1, Q3)	129 (114, 139)	131 (115, 142)	1.656	0.098
BUN, mmol/L, median (Q1, Q3)	6.5 (5.7, 7.2)	6.3 (5.7, 6.9)	1.364	0.173
Creatinine, μmol/L, median (Q1, Q3)	86 (78, 96)	86 (78, 97)	1.102	0.270
Total bilirubin, μmol/L, median (Q1, Q3)	12.9 (9.7, 16.9)	13.5 (10.4, 17.3)	1.160	0.246
Albumin level, g/L, median (Q1, Q3)	38.4 (35.0, 43.6)	37.7 (34.9, 43.0)	1.380	0.168
Tumor type			0.245	0.621
Squamous cell carcinoma	1,175 (93.9%)	418 (94.6%)		
Other	76 (6.1%)	24 (5.4%)		
Primary tumor site			1.188	0.552
Upper	122 (9.8%)	51 (11.5%)		
Middle	755 (60.4%)	259 (58.6%)		
Lower	374 (29.9%)	132 (29.9%)		
Type of operation			3.125	0.077

Open operation	969 (77.5%)	324 (73.3%)		
Minimally invasive esophagectomy	282 (22.5%)	118 (26.7%)		
Surgery time, min, mean (SD)	271.4 ± 48.3	274.8 ± 47.6	1.257	0.209
Intraoperative bleeding, ml, mean (SD)	212.2 ± 118.1	201.4 ± 105.0	1.697	0.090
Anastomosis			0.668	0.414
Cervical	1,011 (80.8%)	365 (82.6%)		
Intrathoracic	240 (19.2%)	77 (17.4%)		
Tumor size, cm, mean (SD)	3.7 ± 1.0	3.7 ± 1.1	0.723	0.470
TNM stage			0.935	0.626
I	133 (10.6%)	43 (9.7%)		
II	641 (51.2%)	238 (53.8%)		
III	477 (38.1%)	161 (36.4%)		
Tumor stage			2.695	0.441
T1	210 (16.8%)	86 (19.5%)		
T2	364 (29.1%)	129 (29.2%)		
T3	519 (41.5%)	181 (41.0%)		
T4	158 (12.6%)	46 (10.4%)		
Nodal stage			2.137	0.544
N0	389 (31.1%)	154 (34.8%)		
N1	524 (41.9%)	176 (39.8%)		
N2	205 (16.4%)	67 (15.2%)		
N3	133 (10.6%)	45 (10.2%)		
Perioperative blood transfusion			0.078	0.780
Yes	275 (22.0%)	100 (22.6%)		
No	976 (78.0%)	342 (77.4%)		
LN harvest, mean (SD)	34.1 ± 12.1	35.3 ± 12.6	1.788	0.074
Postoperative major complications			0.448	0.503
Yes	209 (16.7%)	80 (18.1%)		
No	1,042 (83.3%)	362 (81.9%)		
Postoperative hospital stay, d, mean (SD)	8.8 ± 1.2	8.9 ± 1.4	1.569	0.117

BMI, body mass index; ASA, American Society of Anesthesiologists; FEV_1, forced expiratory volume in 1 second; FVC, forced vital capacity; WBC, white blood cell; LN, lymph node.

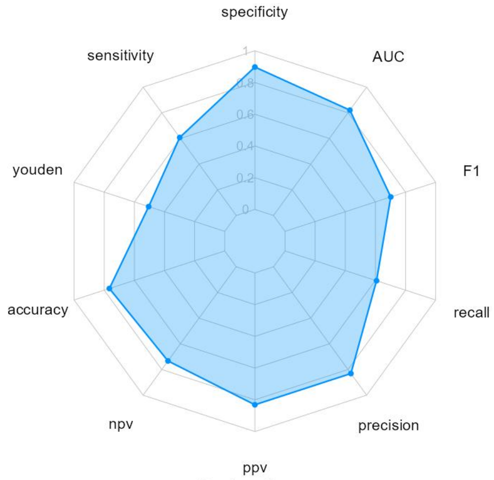

A Random forest

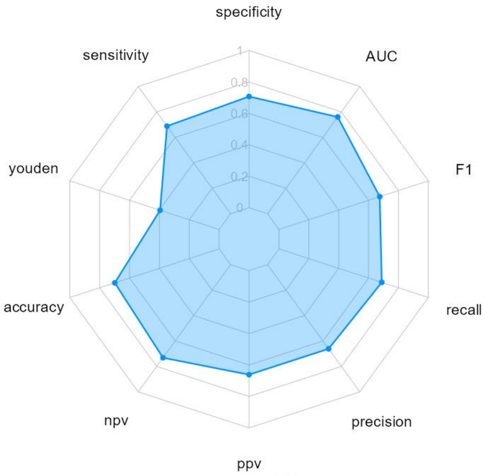

B K nearest neighbor

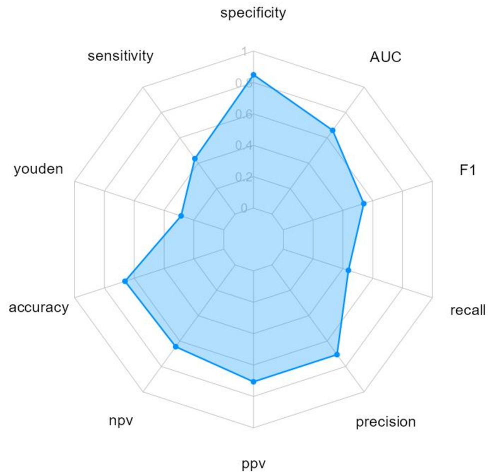

C Gaussian Naive Bayes

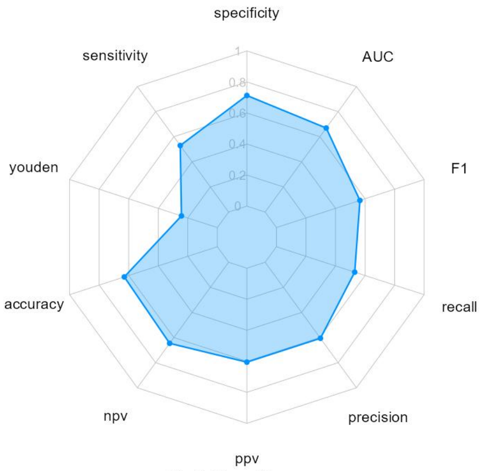

D Partial Least Squares

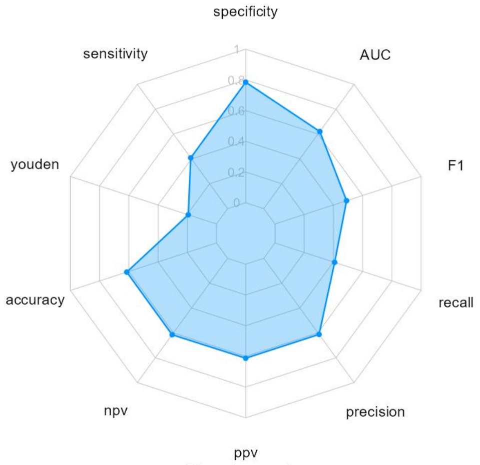

E Neural network

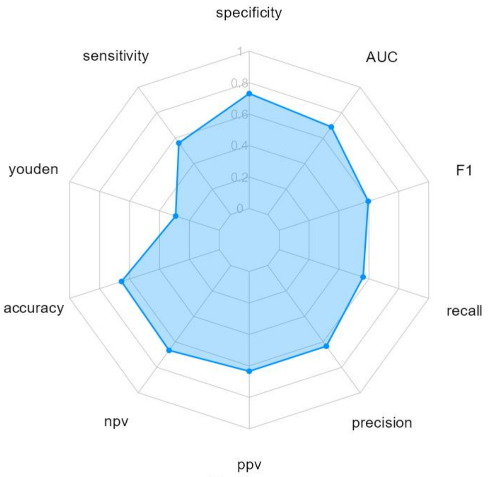

F TreeBagger

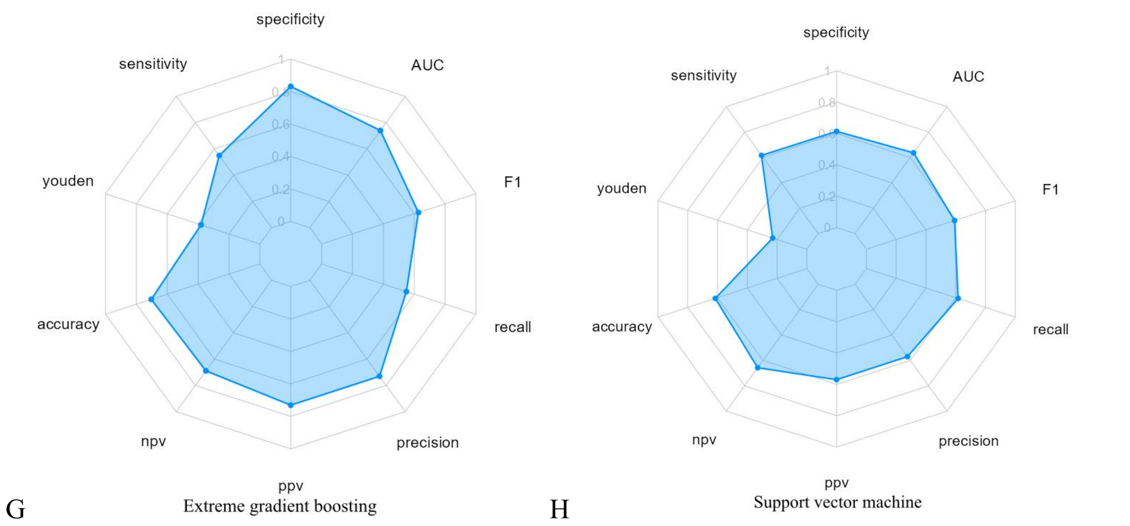

SUPPLEMENTARY FIGURE 2

The radar chart of the development cohort. (A) Random forest, (B) K nearest neighbor, (C) Gaussian Naive Bayes, (D) Partial Least Squares, (E) Neural network, (F) TreeBagger, (G) Extreme gradient boosting, (H) Support vector machine.

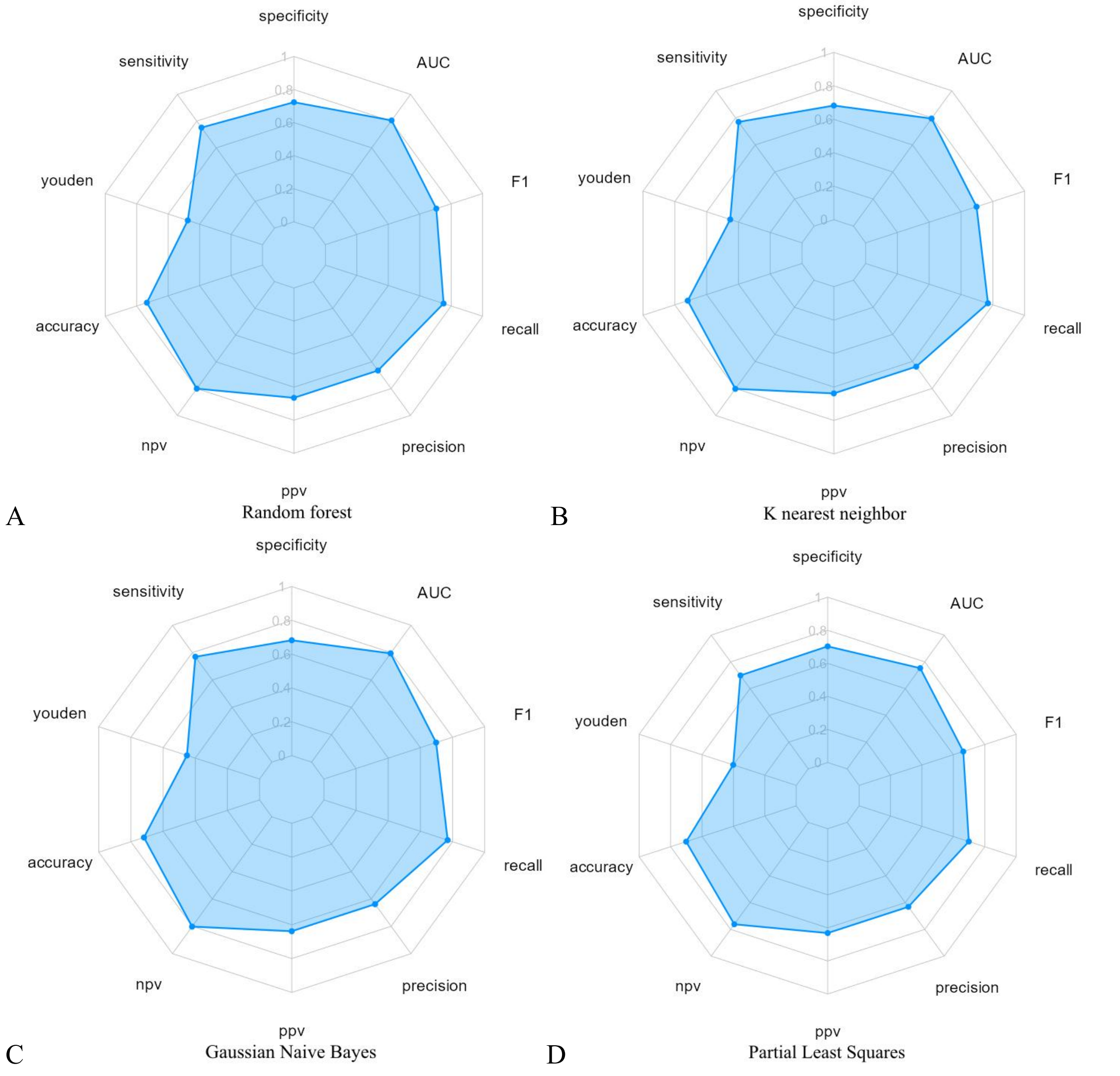

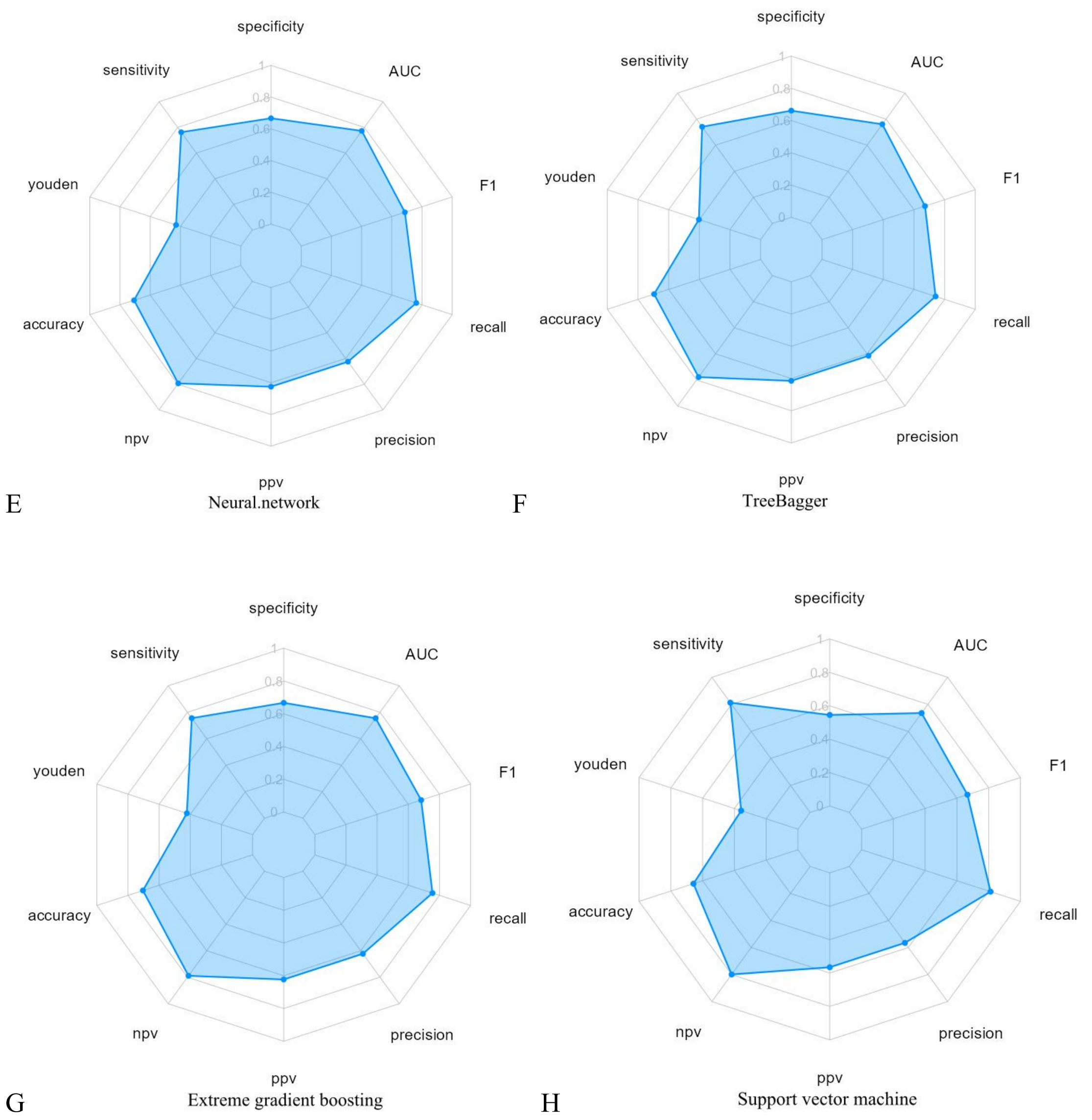

SUPPLEMENTARY FIGURE 3

The radar chart of the validation cohort. (A) Random forest, (B) K nearest neighbor, (C) Gaussian Naive Bayes, (D) Partial Least Squares, (E) Neural network, (F) TreeBagger, (G) Extreme gradient boosting, (H) Support vector machine.

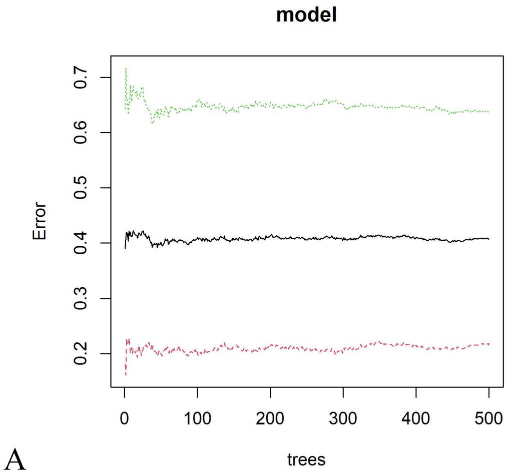

A

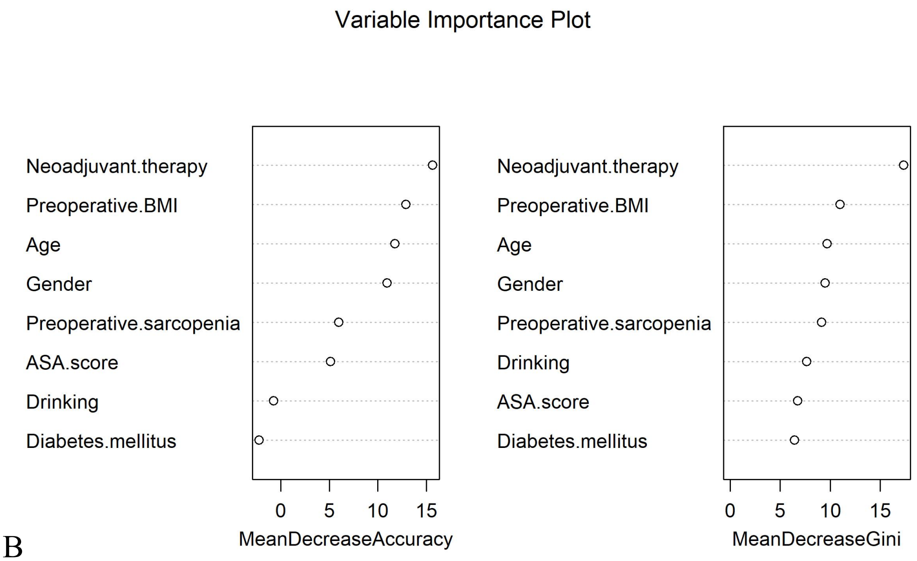

B

SUPPLEMENTARY FIGURE 4

Random Forest Prediction Model. (A) Random forest model; (B) Feature importance ranking.

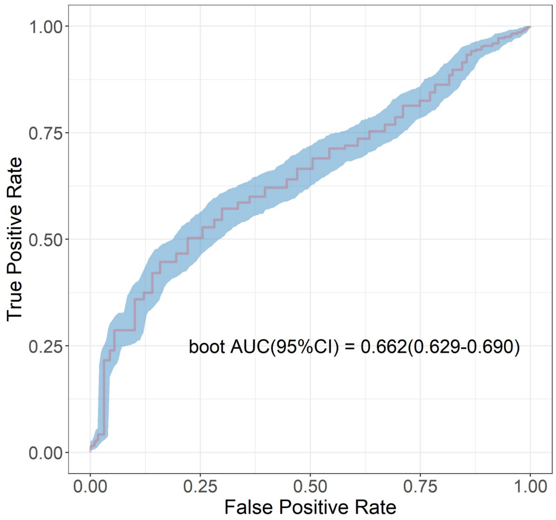

SUPPLEMENTARY FIGURE 5

ROC for the malnutrition prediction model in the age.

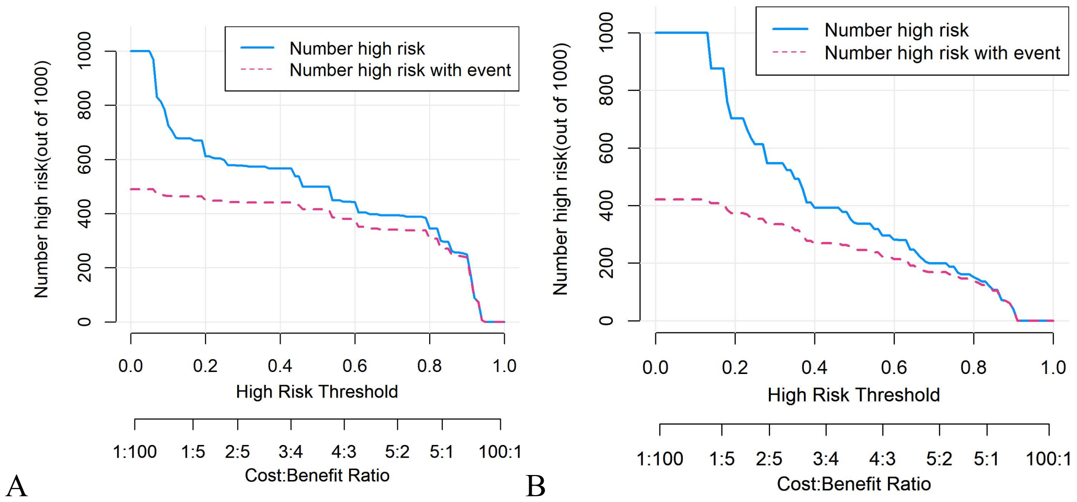

SUPPLEMENTARY FIGURE 6

Clinical impact curves (CICs) for the malnutrition prediction model. **(A)** Development cohort; **(B)** Validation cohort.
